# Knowledge Mobilized in Teacher–Student Interactions in PE in Difficult Vocational High School Classes: Enacted Knowledge

**DOI:** 10.3389/fpsyg.2021.664677

**Published:** 2021-07-14

**Authors:** Amélina Girard, Olivier Vors

**Affiliations:** ^1^ACTé, University Clermont Auvergne, Clermont-Ferrand, France; ^2^Aix-Marseille University, CNRS, ISM, UMR 7287, SFERE-Provence, Marseille, France

**Keywords:** enactive knowledge, enactive phenomenological approach, teacher-student interaction, difficult student, experience, teacher and student voices

## Abstract

**Introduction:** Our study aims to analyze the enactive knowledge mobilized during teacher–student interactions in physical education lessons with difficult classes in vocational high school. These classes are considered “difficult” because they concentrate a large number of pupils who are referred to them because they have no choice. This lack of choice makes it difficult for these pupils to engage in school work and is the cause of deviant behavior and school dropout.

**Methods:** This study was conducted within the methodological and theoretical research program of the course of action. We analyzed the individual activity of nine teachers and 18 students during a PE lesson by collecting audiovisual data and conducting self-confrontation interviews. These data were processed in several stages: transcription, identification, and typification of the components of the experience.

**Results:** The results show mutual active knowledge between the teacher and the “difficult” students: emerging from the context, anchored in a dynamic of experience, coupled with the concerns of actors. The teacher classifies the profiles of students according to their reaction to authority and their difficulties, by spotting students' “thermometers” of the class climate. Some of the knowledge of students is coupled with their concerns to avoid boredom, to avoid trouble with the teacher, and to avoid trouble with their friends.

## Introduction

“What is that teachers need to know if they are to help students make sense of the world in the early 21st century?” This question, posed by Holden and Hicks (2007, p. 13), has been asked for the past 20 years or so, and has been the subject of research in the field of educational science on teacher knowledge (Zembylas, 2007; Craig et al., 2018; Deng, 2018). Shulman (1987) classification provides an institutional view of the knowledge and skills specific to teaching. Since this seminal work, other concepts have made it possible to broaden reflection on teaching according to its pedagogical aspect: content knowledge (CK), general pedagogical knowledge (GPK), pedagogical content knowledge (PCK), knowledge of learners and their characteristics, and knowledge of educational contexts. Grossman and Richert (1988) defines teacher knowledge as a body of professional knowledge consisting of knowledge about broad pedagogical principles, skills, and knowledge about the subject matter being taught. Much research has focused on different types of knowledge in teaching, teacher pedagogical knowledge (for a review, see Depaepe et al., 2013), emotional knowledge (e.g., Zembylas, 2007), contextual knowledge (Tang et al., 2016), or embodied knowledge (Craig et al., 2018). Despite a variety of recent studies, the study of contextual knowledge in teaching contexts is rare, especially in the specific context of difficult classrooms. The difficult educational context in question here is that of the classes in priority education areas within French vocational high schools. In fact, some vocational classes in high schools fail to attract and are therefore attended by students who have not obtained their first choice of orientation (Caille, 2014). These students most often have social and academic difficulties that distance them from the academic norm (Jellab, 2017). Thus, this nonchoice of orientation increases the distrust between students and the school system. Indeed, forced orientation is the primary cause of deviant behavior and school dropout among pupils (Arrighi and Gasquet, 2010). The accumulation of difficulties in engaging in school work for these students makes the classroom context a difficult one for teaching. Our study analyzes the enactive knowledge mobilized during teacher–student interaction in physical education courses with “difficult classes” in vocational high school.

### Knowledge in a Teaching Context

#### Pedagogical Knowledge: From CK to GPK

*Content knowledge* is and has always been intimately linked to the teaching profession and, according to Shulman (1987, p. 9), refers to “the amount and organization of knowledge *per se* in the mind of the teacher.” *Content knowledge* is the teacher's own knowledge of the essential concepts, principles, and modes of inquiry of an academic discipline (Schwab, 1964). CK is at the heart of the teaching content of the teacher. Prior to Shulman's work, *content knowledge* alone defined the tools needed for teaching. The various classifications since Shulman (Grossman and Richert, 1988; Magnusson et al., 1999) have retained CK while supplementing it with pedagogical and institutional knowledge. Although it is no longer presented as the only element of the professionalization of teaching, CK is still very present in educational research. For example, CK is related to the knowledge of learning strategies of students (Loewenberg Ball et al., 2008). The idea is to understand the links between the transformations in the CK initially learned by the teacher to help students learn (Deng, 2018). However, CK is not enough to understand the complexity of learning within the class (Loewenberg Ball et al., 2008).

The notion of PCK was introduced by Shulman (1987) in response to a lack of research in teacher and teaching education that hitherto focused on CK in the United States in the 1980s. Through PCK, Shulman intends to professionalize the teaching profession. Deng (2018) shows that the shift from CK to PCK is linked to the transmission necessary “*to penetrate into the essence of content and to help students grasp the content and develop intellectual and moral powers through encounters with the essence.”* PCK allows a shift from an exclusive focus on CK to taking account of learning difficulties and the related teaching strategies of pupils (Wilson et al., 1987). PCK is a concept connecting CK with pedagogy (Sibbald, 2009). So, PCK becomes the norm of the skill of teachers (Tirosh et al., 2011). However, pedagogical knowledge does not cover all the professionalization of the teaching profession (Depaepe et al., 2013), such as its contextual dimension (Loewenberg Ball et al., 2008). Depaepe et al. (2013) modify the initial model of PCK, adapting it to each intervention context. PCK then becomes “situated.” In the Mathematics context, these authors develop the concept of *mathematical knowledge for teaching* (MKT), crossing CK and PCK.

This importance of pedagogical matters can also be found in the concept of *general content knowledge* or classroom management knowledge. Initially present in Shulman's classification (e.g., Table 1, Summary table of classification of knowledge by Shulman), *general content knowledge* (GPK) is defined as the “*broad principles and strategies of classroom management and organization that appear to transcend subject matter”* as well as “*knowledge about learners and learning, assessment, and educational contexts and purposes”* (Shulman, 1987, p. 8). This concept is used in the current literature (e.g., König et al., 2014; Blömeke et al., 2016) as “*broad principles and strategies of classroom management and organization that appear to transcend subject matter.”* This type of teacher knowledge is then similar to what can be called *classroom management*. Knowledge in terms of classroom management then appears to the teacher as variable cognitive skills, adaptable to a specific classroom context, whereas *CK* would be more stable knowledge for teachers. Classroom management knowledge or GPK, focused on perceiving, interpreting, and making decisions regarding classroom management, is then knowledge that teachers acquire through experience and expertise (Blömeke et al., 2016). This knowledge then enables the teacher to analyze a context and to generate responses appropriate to this classroom context. This knowledge seems to be complementary to CK and PCK in the sense that it allows the teacher to adapt to his or her audience in order to be able to teach. The study by König et al. (2014) shows that GPKs are acquired through the experiences of teacher and are constantly transformed and adjusted. Nevertheless, the authors assume that this knowledge (response pattern) is sufficiently internalized by the teacher to meet the needs of students. In other words, the encounter of several similar teaching contexts allows the teacher to create a repertoire of responses in terms of class management to meet the needs of the students.

**Table 1:** Summary table of classification of knowledge by Shulman (1987).

**Type of knowledge**	**Definitions (key concepts)**
Content knowledge (CK)	Subject matter knowledge
General pedagogical knowledge (GPK)	Subject matter knowledge Strategy of classroom management Knowledge about students, learning, assessment, and educational contexts.
Pedagogical content knowledge (PCK)	Knowledge of different learning strategies Knowledge of potential difficulties encountered by learners Adaptation of subject content to the needs of the students
Knowledge of learner and their characteristics	Knowledge about students in general, and specifically those in the class: importance of context. Knowledge of the psychology of student learning and development
Knowledge of educational context	Classroom knowledge Knowledge of educational policies

#### Situated Knowledge: Between Context Knowledge and Embodied Knowledge

Contextual awareness has emerged in the literature as a helpful tool for analyzing knowledge in teaching contexts. Tang (2003) presents three facets of the teaching context to be taken into account by the teacher: “the action context, the socio-professional context, and the supervisory context.” Knowledge of these different contexts is acquired in the field where the teacher learns to know his or her context of intervention (Ben-Peretz, 2011). Knowledge about the context of intervention is part of the dynamics of construction and development of the knowledge of the teacher. This knowledge seems to directly influence the actions and relationships of the teacher with the school staff (colleagues and hierarchy). The knowledge of the teacher about the context of intervention seems all the more important as there may be cultural dissonance between the teacher and his or her students through different socialization. Tye (1999) stresses the importance for the teacher to be able to understand the context by being able to “see the world through the eyes of others.” This ability to understand the context then enables the teacher to adapt his or her pedagogy to be understood by the students.

The interest of contextual knowledge seems to us to be more important in our context of study: In teaching PE in a difficult environment, the knowledge mobilized by the teacher must be multiple in order to be able to adapt his teaching to this public at odds with the school norm. To our knowledge, little research has focused on the knowledge mobilized by teachers and students in difficult environments (e.g., Vors and Gal-Petitfaux, 2015). This intervention context is problematic in its specificity, which stems from the difficulties in engaging the students in a learning context. These difficulties of pupils present themselves in particular as a difficult relationship with the school rules. Their social origin and educational difficulties make these students have a distant relationship with the school and be “difficult” in the face of the authority that the teacher represents. Their often bumpy routes through it lead them to distance themselves from the school, especially from teachers who represent authority (Jellab, 2017; Kirk, 2019). This distance is most often expressed in a refusal of authority and frequent conflicts between the teacher and his or her students. The specific nature of this context of intervention then raises questions about the knowledge mobilized by the teacher to engage the students without entering into a permanent conflict, a rupture. But also, what knowledge these students mobilize in order to be able to invest themselves in learning and succeed? This line of our research is all the more interesting as recent studies (Vors, 2016) show that students, during PE, become involved by undergoing an alternation of concerns which oscillates between having fun with their friends and responding to the requests of the teacher. The various studies on teacher knowledge in relation to context show that there is a variation in the knowledge mobilized by the teacher depending on the context (Glogger-Frey et al., 2018). Thus, this alternation of concerns in the student generates a particular adaptation and intervention strategy on the part of the teacher, so it seems coherent to us to focus both on the knowledge mobilized by the teacher and that mobilized by the students. Teachers build up a repertoire of knowledge from their initial training in relation to their experience (Harr et al., 2014). Ashe and Bibi (2011) show in their study that the activation of mobilized knowledge depends on the context of intervention. Thus, if we consider the classification made by Tang (2003) of the different contexts influencing the knowledge mobilized by the teacher—the context of action, the socio-professional context, and the context of supervision—our research then focuses on the impact of the context of the action of a “class in a difficult environment,” in relation to the socio-professional context; in other words, our research targets the knowledge that is activated, mobilized, by the actors in the context of teaching in PE.

The phenomenological approach sheds new light on the knowledge of teachers through what the authors call “embodied knowledge” (Parviainen and Aromaa, 2017; Craig et al., 2018). This knowledge is defined as “knowledge incorporated not just by the material body but by a being comprising mind, body and environment” (Gieser, 2008, p. 303). Embodied knowledge then appears as embedded knowledge, marked corporally and emotionally. This knowledge develops through the experience of the individual *via* socialization (cultural and social environment) (Downey, 2010). In this sense, this type of embodied knowledge complements a classification of the knowledge of the teacher which until now was supposed to be acquired through formal learning from continuing education and purely cognitive. Embodied knowledge, therefore, feeds into the knowledge repertoire of teachers by associating bodily experiences with intuitive practices. Embodied knowledge seems to be particularly relevant to teaching through the body: Applied Arts, Music Education, and Physical Education. Parviainen and Aromaa (2017) show in their study on the body knowledge of coach that running training allows the acquisition of sensitive knowledge for the athlete: Through the experiences of the body and by guiding the coach to orient the athlete to bodily feelings, the athlete develops bodily and emotionally marked knowledge. These same authors draw a parallel with PE teaching, questioning how knowledge about the body is produced and transmitted in school. They then consider that it is essential to take into account the body as a source of knowledge for both the student and the teacher. In other words, the teacher constructs a repertoire of knowledge about the body expression of student, and the student, through lived experiences, integrates new, emotionally marked knowledge (Ignatow, 2007). According to Craig et al. (2018), embodied knowledge contributes to the development of the pedagogical relationship, the learning and teaching process.

Zembylas (2007) points to a gap in the literature on *PCK* in educational science research: The emotional side of this knowledge does not seem to be studied and yet seems essential in a profession that interacts with humans. For Zembylas, teacher knowledge is a form of *knowledge ecology*, “a system consisting of many sources and forms of knowledge in a symbiotic relationship: CK, pedagogical knowledge, curriculum knowledge, knowledge of learners, emotional knowledge, knowledge of educational values and goals, and so on.” They then all interact together to enable the teacher–student relationship; in this sense, it seems indispensable to take emotional knowledge into account. The teacher and students develop knowledge about each other or about the discipline in order to understand each other, which Denzin (1994) calls “emotional understanding.” This knowledge is built up through shared experiences in the classroom context. In this sense, we are all the more interested in this knowledge because it integrates teacher–student interactions and the co-construction of mutual knowledge in order to be able to teach/learn in a particular social and environmental context.

#### Enactive Knowledge: An Enactive Phenomenological Approach

The foregoing literature review invites us to consider teacher knowledge beyond Shulman (1987) initial classification. The knowledge of the teacher is not fixed; it evolves and is updated. The teacher then creates a repertoire of knowledge both through experience (Harr et al., 2014) and according to the context in which he or she works (Glogger-Frey et al., 2018). This research then leads us to consider knowledge in the context of teaching in the framework of enactive phenomenology in order to understand more precisely how it is constructed and updated (Sève et al., 2002, 2005; Theureau, 2015). Enactive phenomenology considers that all human activity leads to a process of learning and development. This learning and development process underlies the idea that all activity of an actor mobilizes knowledge. Knowledge is experiential, that is to say, dependent on the interaction of the actor with his/her environment at an instant t. This framework considers that experience is made up of significant interdependent components such as action, perception, concern, and knowledge. The constant interaction between the actor and the environment forms an autonomous system in permanent transformation (Maturana and Varela, 1980). Therefore, knowledge exists only in and through experience, in the interface of the material and human environment, concerns, and culture of the actor. Experience stems from the dynamics of the actor–environment coupling; this coupling gives rise to what Rosch calls types (Rosch, 1973). Types are knowledge categorized by traits of similarity or elements of generality, which make it possible to make the link between past and current experience by validating or invalidating “type knowledge” or by constructing new knowledge through the perceptions of the actor or by linking already-existing types (Rosch, 1973; Sève et al., 2002). In this framework, when considering the knowledge emerging from the activity of the actor in context, the latter cannot be isolated from the components of the experience. This concept of “type” is used in the field of PE (Guillou and Durny, 2008) and sport (Sève et al., 2002, 2005; Bourbousson et al., 2011). For example, Sève et al. (2005) showed that the knowledge of high-level sport players was continuously related to significant elements in the unfolding situation in order to create interpretive chains, and this contributed to building a significant world for the players. Knowledge is intertwined with the perceptions and concerns of the player in the context that generate the mobilization and/or updating of knowledge by the player in the action and in relation to the frame of reference, i.e., the solicitation of part of the culture of the player and elements of generality from his/her past courses of action. In other words, apprehending knowledge in a teaching context through an enactive phenomenological anchoring means understanding the interdependence of the components of the courses of action of actors in order to categorize the mobilized and/or updated knowledge according to the typical concerns or those with similarities and the referential of the actor in activity.

Thus, in a phenomenological approach to understanding the knowledge brought into play in a teaching context, we can then consider that this knowledge is enacted, that the knowledge emerges and is inseparable from the context. Enactive knowledge means considering that knowledge in a teaching context is constantly being redefined and adapted to the environment. The accumulation of experience within the classroom then allows the actor to create a repertoire of knowledge that responds to typical concerns in contexts encountered according to their similarities. Apprehending knowledge in a teaching context as enacted leads us to invest, *in situ*, the knowledge created and mobilized in a classroom context, more precisely in PE within difficult classes. So, our study seeks to analyze the enactive knowledge mobilized during teacher–student interaction within physical education courses with “difficult” classes in vocational high school.

## Methodology and Methods

### A Specific Difficult Context

Case studies are conducted in three vocational high schools. Vocational high schools in France are special. Teachers have to cope with a higher rate of violence, misbehavior, truancy, and dropout than in other schools (Jellab, 2017). In this context, teacher–student interactions can be particularly problematic. Nine teachers (three per school) are volunteers to be participants of the study, with an age range of 23–55 years [22.2% women; mean age (M) = 35.96, standard deviation (SD) = 10.29]. And they were chosen because they were considered “successful teachers” (Chauveau, 2001; Vors and Gal-Petitfaux, 2015; Vors et al., 2015): considered to be efficient with “difficult” students in terms of classroom management in particular. Eighteen students are chosen by the teacher (each teacher chooses two students considered the most difficult), with an age range of 15–19 years [18.5% girls; mean age (M) = 16.23, standard deviation (SD) = 0.87]. The selected classes are considered the most “difficult” of the high school by the teacher. In these classes, during the study, there are five students expelled from school, 10 subjects to Disciplinary Board, and a lot of violence between students (e.g., more than one fight per week) or against the teacher (e.g., a student throws a stone at his head of teacher). The teacher team of this class considers that interactions in the class are difficult, due to the frequency of misbehavior, and linked to the sensitivity of the students (in opposition with school, refusal of authority). In that context, the question of enactive knowledge seems to be central. How do teacher and students manage these difficulties? What knowledge helps them to act in the class?

### Data Collection to Access the Contextualized Experience of Actor

Two types of data are collected: audiovisual recordings and enactive interviews. Eighteen physical education lessons are recorded by a wide-angle camera and a high-frequency microphone worn by the teacher or the student. Each lesson lasted 90 min of actual teaching. In each of the nine classes, two lessons are collected at the beginning and in the middle of the second term (between January and April), in nine sports (track and field, gymnastic, acrosport, climbing, badminton, table tennis, handball, rugby). These data are used to identify traces of the classroom activity of teachers and students and their interactions. Then, enactive interviews are performed (Theureau, 2003, 2015; Vors et al., 2019). In total, 54 interviews are collected: 18 from the teachers (two per teacher) and 36 from the students (two per student). Generally, the interviews last 30 min with student and 60 min with teacher, in the 48 h following the lessons.

Enactive interviews are a usual method connected to the enactive phenomenological framework (De Jaegher and Di Paolo, 2007; Froese and Di Paolo, 2011; McGann et al., 2013; Theureau, 2015) in accordance with the semiotic approach to cognition and action inspired by Peirce (1978) (e.g., Jourand et al., 2018; Rochat et al., 2018; Dieumegard et al., 2019; Vors et al., 2019). This semiotic approach considers that human experience is composed of significant units like action, concern, perception, and knowledge, as used in this study. Thus, significant experience corresponds to the significant units that can be described, commented on, and shown by the actor. In this way, we may access experience of actors through its significant dynamic (Varela et al., 1992). From this perspective, the signification of the actor is constructed during the action and can be revealed following a rigorous phenomenological method using a self-confrontation interview known as the enactive interview. A few recent studies have demonstrated the fruitfulness of this method in the fields of education (Dieumegard et al., 2019), stress (e.g., Vors et al., 2018), sport (e.g., R'Kiouak et al., 2018), doping (e.g., Hauw, 2017), and work (e.g., Horcik et al., 2014).

The method of this interview is very directive so as to provoke the re-emergence of elements of past experience when the actor is confronted with his own video recording. Whenever he wishes, the actor can pause the video to describe, comment on, and show his own lived experience step by step. The actor does not know the aim of the experiment. Before each interview, the researcher explains the expectation that the actor should “re-live” and describe his own experience during the PE course, without a posteriori analysis, rationalization, or justification, as suggested for phenomenological research (Starks and Brown Trinidad, 2007). In order to eliminate pre-formed telling, the actor is involved in an attitude of evocation. He is directed to avoid a theoretical description of his action but to evoke what he experienced during the specific moment on video. Behavior indicators like hesitations in the stream of language, unstructured sentences, or an introspective stare are synonyms of evocation (Hauw and Durand, 2008). The starting question about a teacher–student interaction is: “What are you doing here (pointing to the video image moment of teacher–student interaction)?,” then: “What do you perceive at this moment?,” “What are your concerns?” According to the response of the actor, the questioning goes deeper, starting from the evocation of the actor. If an actor emphasized a certain knowledge during the interview, the principle of in-depth qualitative research dictated that the researchers investigate a more explicit and authentic report of the experience, always in relation to the unfolding situation. This qualitative approach produces a different point of view on the classroom knowledge in a difficult context, because we can identify the emerging knowledge during the class context, its evolution, and its relationship with other components of the contextualized experience like perception and concerns.

### Data Analysis

This data analysis of teacher–student interaction was conducted in five steps following the custom of this theoretical and methodological framework (Theureau, 2003, 2015).

First, the 54 enactive interviews were transcribed verbatim and related to the chronological description of actions and communications using audio-video records (e.g., **Tables 7**, **11**).

Second, components of the experience were identified using the verbatims associated with the chronological description: perception (what sensations, sentiments, feelings are significant in the context?), action (what does the actor do?), concerns (what is his intention?), and knowledge (what knowledge emerges within the action?; in other words, what knowledge is mobilized to act?). For greater clarity, we normalized the formulation of the content of each component succinctly. Each component of the experience was reported using emblematic words of the actor (e.g., Table 2, Enactive Interview excerpt as a student).

**Table 2:** Enactive interview excerpt as a student.

**Enactive Interview as Timo (Student)**
[Researcher]: What are you doing here? [Timo]: I don't know. [Researcher]: Does Mr Simon look familiar to you? [Timo]: Yeah. [Researcher]: What's he saying to you? [Timo]: I don't even know anymore. I think he said “You have to help.” And so in the end I went to get a drink and I went to the locker room, I left for some time and I came back. [Researcher]: You're clever, you let it happen! How do you feel about him saying that to you at that moment? [Timo]: Basically, it means “Move yourself!” [Researcher]: It means “Move it!” “What does it mean to you that he's telling you that at that moment?” [Timo]: Since I'm making fun of him, I'll give him something else. [Researcher]: How does he tell you “Move yourself!”? [Timo]: He says, “Go on, Timo, help a little!” or “You have to help,” something like that! [Researcher]: How do you feel when he tells you that? [Timo]: Nothing, it makes me laugh. (Laughs)

Third, the typical relationships between components of the experience were identified at the intraindividual and interindividual level for each teacher (18 enactive interviews) and each student (36 enactive interviews). Typicality corresponds to four aspects (, Durand, 2014; Vors et al., 2019): descriptive, statistical, generative, and significant. Descriptive, because the typical occurrence presents the highest number of traits of the experience analyzed in the sample of actors and the contexts studied. Statistical, because the typical occurrence is the one which is the most frequently observed in the sample studied. Generative, because the typical occurrence has a propensity to recur when conditions resemble those observed are reproduced. Significant, because the actors express that point is highly representative, important during their action. These typical relationships of the experience belonged to the synthesis of the two enactive interviews of the same actor. In each lesson, the intraindividual recurrences are identified and then only the interindividual recurrences are kept. This construction crossed the preceding steps associating temporality, perceptions, actions, concerns, and knowledge. Thus, each typical component of the experience was analyzed for each of the nine teachers and 18 students: to understand the typical relation between knowledge and dynamics of action, between perception and knowledge, then between knowledge and concerns.

Fourth, the typical relationship between components of the experience for all the teachers and for all the students was identified by comparing all the individual typical experiences (step 3). Data were compared to keep only the typical component of the experience recurrent for all.

Fifth, we constructed the tables of typical knowledge linked with other components of the experience for the nine teachers and 18 students (e.g., Tables 3, **8**).

**Table 3:** Contextual knowledge observed in relation to contextualized perceptions.

**Meaningful perception for the teacher**	**Knowledge mobilized in action by the teacher**
Student who keeps his jacket on at the beginning of the class	Student is not ready to engage in the situation.
Student jumping on the mats while waiting for the start of the class	Student is impatient, he must be channeled by explaining the course to him.
Student hiding	Student refuses to get involved in the situation, you have to position yourself close to him/her.
Student approaching the teacher to work	Student demands attention, his or her work must be valued by additional instructions.
Student who questions the teacher about practice	Student is worried about the expectations of the situation; he needs to be reassured by explaining the criteria for success.
Student who does not practice	Student does not want to show that he does not master what is expected, he must be encouraged. Or Student has an unusual problem, he must be questioned in order to show that he is interested in him.
Student who behaves in an off-task manner	Student wants to have fun, you have to try to understand why in order to intervene in an appropriate way.

## Results: the Classroom Activity Organized by Enacted Knowledge

There is a constant agitation during the classes studied with numerous problems of violence and misbehavior. At the beginning of the PE course, students come out of the changing room shouting and running. The teacher is waiting in the gym. He asks the students to sit down in front of him. Then he quickly gives the instructions of the day for few minutes. During the exercise, there is a lot of noise, but the majority of the students are in the working zone trying to do the exercise. In spite of the potential level of agitation of the class, the teacher manages to put his students to work. This example of a usual beginning of lesson demonstrates the potential of these difficult classes within the vocational high schools studied. However, the results show a work climate during the lesson. That work climate in the class is induced by the mutual knowledge between the teachers and the students. The enacted knowledge of the teacher about the students and that of the students about the teacher is particularly significant in their experiences *in situ* and organizes their actions.

### Teacher Results: An Intervention Based on In-Depth Knowledge of the Students

The analysis of the activity of the teacher *in situ* shows that the knowledge he or she mobilizes emerges from a significant context related to different components of the experience of the teacher as well as his or her concerns. In order to intervene in an appropriate manner with regard to their particularly “difficult” students, teachers mobilize enacted knowledge: contextual, dynamic, and coupled with their concerns.

#### Mobilization of Contextual Knowledge in the Classroom to Interpret the Activity of Students

The results show that teachers mobilize contextual knowledge in action according to their perception of the context. Depending on the significant elements that the teacher perceives of the activity of students in context, he/she mobilizes certain knowledge enabling him/her to intervene in context (Table 3).

Table 3 synthesizes the typical knowledge of teachers faced with “difficult” students, that is to say the most characteristic of the activity of teachers, the most frequent in the different lessons studied, and common to the different teachers studied. The perceptions of the teacher update his/her knowledge allowing him/her to interpret the context *in situ* and to know how to intervene in an appropriate way. In other words, the teacher mobilizes his/her contextual knowledge in order to analyze the commitment of the students according to what he/she perceives of their activity; this knowledge enables him/her to intervene in an appropriate way with these “difficult” students. This knowledge only makes sense in the context of the situation: if we consider the following example, the teacher analyzes the off-task behavior of students in a context of the beginning of the class (Table 4).

**Table 4:** Excerpt from enactive interview teacher.

**Actions and communications**	**Active teacher interview**
*Second floor gymnastics lesson. The teacher announces to the students that they have to do gymnastic sequences like last week.Ousmane and Fleurcy start singing.The teacher observes them and then intervenes by reminding them of the instructions: “Try to occupy the whole space by jogging without worrying about others.”*	*I look at Ousmane and Fleurcy, um. they are singing. I wonder if they are in it or not. I look at the elements that allow me to know if they are engaged (in the work) or not. And in fact they are in it because they are singing the music of the challenge (in gymnastics). I was ready to tell them “no, guys, we don't sing,” then I say to myself in my head: they seem to be rather in the session, I mustn't reprimand them now, otherwise they'll get up in arms and say “Sir, I was there, I'm there.” So I look over there, and I'm just going to remind them of the instructions on how to occupy the space*.

This example shows us how the activity of the teacher is organized with difficult students singing. Thanks to his knowledge of the context, he knows how to interpret the behavior of students. Here, the teacher interprets that Ousmane and Fleurcy are engaged in the requested work, even if they are singing. He understood that the song was related to the music of the gymnastics challenge of the previous week. His knowledge of the music of these students constructed the week before allows the teacher to understand that the students are engaged. Moreover, thanks to his in-depth knowledge of his students, he adapts his interventions. The teacher knows that Ousmane and Fleurcy are difficult, and they are hyperactive and react badly to the reprimands of the teacher. He, therefore, chooses to intervene indirectly by reminding them of the instructions on how to occupy the space.

The knowledge mobilized by the teacher is therefore contextual; it is articulated according to perceptions in action and guides his or her intervention. We shall see that the knowledge of the teacher is also anchored in a temporal dynamic.

#### Knowledge of Student Profiles Rooted in a Dynamic of Experiences

Our results show that teachers build up a detailed knowledge of the profile of certain students, enabling them to adapt their interventions to difficult classes. The knowledge of teachers is mobilized and updated in action. The knowledge they have of particular students is anchored in the dynamics of their experiences. The detailed knowledge of teachers, therefore, refers to previous experiences with their students. Each context experienced in the classroom participates in the construction or updating of their knowledge. The accumulation of experiences allows the teacher to build a repertoire of knowledge categorizing the students according to different profiles. The analysis of these different types of knowledge has made it possible to identify the main categories emblematic of the knowledge of the students that teachers have with their difficult classes (Table 5).

**Table 5:** Knowledge of student profiles in relation to the type of intervention appropriate to them.

**Knowledge about student profiles**	**Appropriate type of intervention**
Relation to authority: Open student: “able to understand,” not resentful Closed student: proud, refusal of authority.	Intervene on these students to regulate the activity of others. Avoiding conflict: challenge strategy, indirect intervention.
Student “thermometer” of the class: Group leaders Student to regulate the intensity of the lesson	To encourage and value these students in order to engage others. Regulate the task when these students drop out.
Difficulties in discipline: Student with motor difficulties Student who tries hard Students capable of success but afraid of failure Students at ease with their motor skills	Giving these students the confidence to invest in themselves Encouraging and congratulating these students for continuing to work Challenging these students to dare to try Individualizing the instructions to go further in the requirements

Table 5 allows us to categorize the fine knowledge of students that teachers have in the form of a typical profile. The typical profiles retained during the analysis are those that are the most important from the teachers' point of view, the most frequently mobilized in the different lessons studied, and those that are common to the different teachers studied. This knowledge of the students is built up as they interact with each other. From the beginning of the year, the teacher is very attentive to the reactions of students in class. For example, Teacher D constructs the knowledge that Soufiane is a “closed” student refusing authority as early as the first session, when he perceives that this student withdraws after a reprimand. In the following session, the teacher intervenes indirectly to put him/her at ease; from the beginning of the lesson, he discusses with him/her about his/her activities outside the school. This anecdote is one example among others showing that knowledge is built up as the classroom experience of the teacher unfolds.

#### Knowledge Coupled With the Concerns of Teacher

Our results show that teachers mobilize knowledge in the classroom in relation to their dominant concern in the context. The analysis highlights the two dominant concerns of the teacher with difficult classes: “(Re-)engaging in the situation” and “exercising authority while avoiding conflict.” The *in situ* intervention of the teacher is based on knowledge coupled with his/her dominant concern at a given moment t (Table 6 Knowledge mobilized by the teacher to satisfy his or her dominant concerns).

**Table 6:** Knowledge mobilized by the teacher to satisfy his or her dominant concerns.

**Overriding concern**	**Coupled knowledge**
(Re)engage the student	Giving precise individual instructions by means of roll calls or injunctions Use the non-verbal: physical contact (e.g., hand on shoulder), getting closer to the student, making gestures (e.g., pointing), insistent glance Challenging the student Discussing with the student Taking an interest in the student
Exercising authority while avoiding conflict	Keep calm, do not respond to provocations, do not show your emotions. Isolate and intervene face to face. Show yourself. Demonstrate understanding of the student. Acting in a roundabout way (e.g., intervening on another student to set an example). Focusing on working students.

Table 6 synthesizes the knowledge mobilized by teachers according to their two dominant concerns. These couplings between knowledge and concerns are typical because they are characteristic from the teachers' point of view, they are the most frequent in the different lessons studied, and they are common to the different teachers studied. The originality of our results is to show that according to their concerns, teachers adjust their modalities of intervention by mobilizing appropriate knowledge. The following extract shows an example where the knowledge mobilized by Teacher J is coupled with his concern to engage Ismaël in the task (Table 7).

**Table 7:** Excerpt from Teacher J, session 6.

**Actions and communications**	**Active teacher interview**
*In gymnastics, the students have to cross the mats in waves by rolling forward.Ismaël did not join the first wave.The teacher calls out to him and says, “I know you, Ismael, I know you….”*	I see that Ismaël doesn't do the exercise. It's important not to let this happen, otherwise the whole class will gradually do nothing. I tell him directly: “*I know you, Ismaël, I know you,”* You have to say it quickly and immediately for it to have an effect. Now I want to challenge Ismaël, that's how it works with him. I've experienced it in basketball. I want to provoke him, he is a sportsman who does well, he has the means to succeed. […]. “I know you” means “I know that you are capable” and also “I've seen you.” I want to make him react, to challenge him. Otherwise, with him, it's certain that he won't do anything, he doesn't want to show that he's in trouble. He prefers to do nothing rather than show that he is in difficulty.

This example shows us the knowledge that the teacher mobilizes to engage Ishmael in the rolling task. Engaging the students is the dominant concern for Teacher J at this point in time. To engage Ismaël, the teacher mobilizes various knowledge that he has built up previously: he knows that he has to intervene quickly, that he has to challenge him in order to prevent him from dropping out in a lasting way, he knows that Ismaël is capable of doing the exercise.

We have thus seen that intervening with difficult classes is a complex activity involving many components of the experience of the teacher. The originality of our study is that it shows that teachers have a detailed knowledge of their students which enables them to adapt their interventions. This knowledge is valid insofar as it emerges from a situated context, is dynamic, and is linked to the concerns of teachers.

### Student Results: Student Activity Based on a Detailed Knowledge of the Teacher

The analysis of the activity of the 18 “difficult” students studied shows that the knowledge they mobilize emerges from a significant context related to different components of their experience. In order to take action, the student mobilizes enacted knowledge: contextual, dynamic, and coupled with his or her preoccupations.

#### Mobilization of Contextual Knowledge in the Classroom to Interpret the Activity of Teacher

The study of the activity of the “difficult” students in the PE lesson shows that the student mobilizes knowledge *in situ* according to the elements that are significant for him/her in the context. What the student perceives from the context generates the mobilization of knowledge for action. The most significant elements for the student are related to the actions of the teacher. During our study, we identified the contextual knowledge of students that enables them to interpret certain actions of the teacher (Table 8).

**Table 8:** Contextual knowledge of the student.

**Meaningful perception for the student**	**Knowledge mobilized in action by the student**
The teacher looks at me insistently, frowning.	The teacher is angry, he's watching me, it's better to work.
The teacher is close to me. The teacher is behind me.	The teacher is watching me, I can't be off-task.
The teacher turns his back on me.	The teacher doesn't look at me, I can have a little fun.
The teacher calls me by name, challenging me.	The teacher is interested in me, he wants to “boost” me, I have to show him what I am capable of.
The teacher points at me. The teacher says my first name. The teacher touches my shoulder.	The teacher saw that I wasn't doing the exercise, I have to work.

Table 8 Contextual knowledge of the student synthesizes the typical knowledge of the “difficult” student, that is to say, the most characteristic of the activity of students, the most frequent in the different lessons studied, and common to the different students studied. The data have highlighted a great diversity of contextual knowledge mobilized by different students; however, only five items are typical of the activity of all the students studied. The student mobilizes a precise repertoire of contextual knowledge about the teacher enabling him/her to regulate his/her own activity. For example, in the hubbub of the class, Nicolas hears his first name. Among the different noises, it is his first name which is the significant perception for him at that moment. He knows that the teacher has “caught him red-handed” (i.e., is watching him). Without looking at the teacher, Nicolas goes back to work as if nothing had happened. During the energetic interview, he explains: “There, the teacher spotted me having fun. I pretend I didn't hear him and went back to work as if I had been working from the beginning. I have to show him that I am working, otherwise, I will get into trouble.” This anecdote shows that the knowledge mobilized by Nicolas is contextualized in relation to a perception that makes sense to him. This contextualized knowledge of the actions of the teacher enables him to adapt his actions to “avoid trouble.”

#### Knowledge of the Profile of Teacher Rooted in a Dynamic of Experiences

Analysis of the activity of students in difficult environments shows that each one builds up knowledge about the teacher that is specific to him or her. This knowledge is dynamic and is built up as they interact with the teacher. Thus, over time, the various students in the class categorize this knowledge in order to associate their teacher with a particular profile. Our results show three main teacher profiles derived from the different knowledge in action of the different students studied (Table 9).

**Table 9:** Knowledge of teacher profiles in relation to the type of action adapted.

**Knowledge about teacher profiles**	**Suitable type of action**
Authoritative According to the students, the teacher: “… is always picking on us—Forbidden to have fun—Accuses, with him it's always me who's at fault—Spends his time shouting and punishing.”	Do nothing, stand aside. Have fun making him “freak out” Show the work when it is there. Wait until his back is turned to have a good time. Hide your actions.
Supportive According to the students, the teacher: “…is interested in us—Tries to understand—Discusses with us—Wants us to succeed—Encourages—Praises.”	Discuss with him Working to please him To show him that I am capable, that I make an effort. Trust in us
Capricious According to the students the teacher: “… is inconsistent: one time he says nothing, another time he shouts at us—Change all the time: we laugh with him, then he gives us a bad mark.”	Beware of him Find out if he's having a good day Say nothing to him Laughing with him or avoiding him

Table 9 shows how students categorize their teachers according to three main profiles. However, these categories are not stable because the same teacher may be categorized differently for different students. According to the dynamics of teacher–student interactions, the knowledge constructed is not the same. In interaction, difficult students are particularly marked by the interventions of teachers aimed at building their confidence and managing an incident in the classroom.

Moreover, this knowledge and these profiles are not stable and may vary over time. The knowledge to determine the profile of the teacher is rooted in the dynamics of the experience of students. This knowledge is built up in context and is updated through other contexts with similarities or involving a past event. For example, Mohamed had categorized Teacher W as authoritarian from the beginning of the year because he had punished a student in the first lesson. In subsequent sessions, Mohamed would get up in arms and take the interventions of the teacher as a reprimand. Then in March, Mohamed changed his mind toward the teacher. Two things were particularly significant for him. On the one hand, an informal discussion about fashionable songs he had with the teacher before going into the changing rooms. Second, a challenge the teacher made in badminton. Laughing, the teacher challenged him to break his rally record by telling him that he could do it. These interactions had a special meaning for Mohamed, who no longer experienced the interventions of the teacher as reprimands, but as aids. For him, the perception of the teacher gradually changed and he finally saw him as a supportive teacher who was interested in him. This singular example shows the originality of our research, which allows us to account for the dynamics of knowledge of students.

#### Knowledge Coupled With the Concerns of Students

The knowledge mobilized in context by the student is coupled with his or her concerns of the moment. The analysis highlights the three dominant concerns of “difficult” students: “avoiding boredom,” “avoiding trouble with the teacher,” and “avoiding trouble with one's classmates.” The difficult student's classroom experience is rooted in knowledge coupled with his or her dominant concern at a time t (Table 10).

**Table 10:** Knowledge mobilized by the student to satisfy his or her dominant concerns.

**Overriding concern**	**Coupled knowledge**
Avoid boredom	Having fun when the teacher is away Challenging each other as students Change your exercise often Inventing exercises Getting together with friends Negotiating the rules
Avoid trouble with the teacher	Show the teacher work to be able to play afterwards Avoid being punished Observe whether the teacher is upset
Avoiding trouble with peers	Don't show that I am working Hiding intentions to succeed Don't show weakness Don't let yourself be fooled Don't lose face

Table 10 synthesizes the knowledge mobilized by the student according to his or her dominant preoccupations. These couplings between knowledge and concerns are typical because they are significant from the students' point of view, they are the most frequent in the different lessons studied, and they are common to the different students studied.

However, these concerns are difficult to reconcile, which often places students in dilemmas with contradictory mobilized knowledge. For example, showing work to avoid trouble with the teacher, while hiding their work so as not to lose face in front of their peers. The following excerpt helps to understand the difficult articulation of concerns of students in action as well as the knowledge they mobilize to respond to them (Table 11).

**Table 11:** Excerpt from Ousmane's two-column table, session 4.

**Actions and communications**	**Active student interview**
The teacher waits to give instructions. Ousmane speaks loudly to his comrades next to him. The teacher calls Ousmane “Go Ousmane!” Ousmane moves away from his comrades and returns to work.	[Researcher]: What's going on here? You sound like you're talking loud, don't you? [Ousmane]: They're laughing at me. [Researcher]: They're laughing at you? [Ousmane]: Well, yes, because there they tell me “you're black, with your haircut you look like a sponge.” [Researcher]: Oh yeah? Your friends there? [Ousmane]: Yeah, Fleurcy, Sady and the rest. [Researcher]: Okay. So, what's going on? Ousmane]: I answer, I mustn't let myself be taken in, otherwise I'll look like a weakling, a washout. [Researcher]: What's your answer? I say the same thing, I make fun of them, I speak loudly. [Researcher]: Are you speaking loudly? Ousmane]: I speak loudly to show that I'm not afraid, too bad if the teacher looks at me. Because if I speak softly, they'll say “Yeah, why are you being so smart, you're a sucker, you speak softly and so on and so on.” I showed them what's what. [Researcher]: And the teacher? [Ousmane]: Yeah, it's getting hot, she spotted me. As soon as I'm finished with them, I have to show her discreetly that I'm doing the work.

This excerpt illustrates Ousmane's desire to satisfy various contradictory preoccupations “to avoid trouble with his friends Fleurcy, Sady, and the rest” and “to avoid trouble with Teacher H.” He doesn't want to lose face in front of his friends, who will make fun of him and, at the same time, he doesn't want to be in conflict with the teacher.

To conclude, our results showed the complexity of the activity of difficult students in the classroom in relation to typical enacted, contextual, dynamic, and preoccupation-coupled knowledge.

## Discussion

The originality of our results is that they present mutual enactive knowledge between teacher and “difficult” students: (a) emerging from the context, (b) anchored in a dynamic of experience, and (c) coupled with the actor's concerns.

First, our results have shown that this knowledge is not isolated and cannot be dissociated from the context. This knowledge allows teachers to interpret the action of students in relation to significant perceptions of the context. For example, when the teacher perceives that a student keeps his jacket during the lesson, this updates his knowledge allowing him to interpret that at that moment the student is not ready to engage in the situation. Our results enrich the literature on classroom management knowledge or GPK focus (e.g., Blömeke et al., 2016). This knowledge allows the teacher to perceive and interpret in order to manage the class according to the context. It is more contextualized than CK, which is more general. It is constructed, adapted, and adjusted in experience by encountering several similar teaching contexts. This allows the teacher to create a repertoire of responses in terms of classroom management to meet the needs of the students (König et al., 2014). This experiential construction of a repertoire of knowledge is close to the categorization by “family likeness” specific to our theoretical framework (Rosch, 1973). The significant perception of the actor brings out knowledge which is compared by judging family likeness with knowledge already constructed in other contexts. Moreover, our results are innovative because they also bring out the student voices. They show the knowledge that the students have built up on the teacher to interpret the context. The perception of certain characteristics (action, voice, face) of the teacher is related to a repertoire of knowledge allowing the student to interpret the emotions and concerns of the teacher. This is consistent with some work in the classroom context, showing the importance of emotional analysis in the classroom (Zembylas, 2007; Ruzek et al., 2016). These results could complement the notion of competencies of the entrepreneurial teacher which is defined through “three aspects of entrepreneurial teachers based on their action characteristics: First, entrepreneurial teachers are good at listening and can find good ideas from conversations; second, they are proactive and good at selling their own ideas to others; and third, they cultivate students' enthusiasm for creation, growth, and learning” (Huang et al., 2020). Indeed, the knowledge gained, especially about the behavior of individual students, seems to reinforce the competence of the entrepreneurial teacher in observing and listening to the class. This raises the question of initial training in teacher–student interactions in a difficult context, so as to enable teachers, especially novices, to enrich their repertoire of knowledge for classroom intervention.

Second, the originality of our results is to present classroom knowledge anchored in a dynamic of experience. It is not considered stable and static; it undergoes perpetual adjustment. We have seen that classroom interactions lead the teacher to categorize his/her students by profiles according to their reaction to authority, and their difficulties, by identifying students as “thermometers” of the classroom climate. These results are in line with work showing that the interactions of teachers take place according to the categorizations he/she makes of the students in the class, such as “steering criterion groups” (Dahllöf, 1971; Lefeuvre and Murillo, 2017). These works analyzing teacher–student interactions show that teachers decide and regulate their actions primarily on the basis of the behavior and reactions of a group of a few students. If these students “give signs” indicating that they are following and understand, teachers continue without modification. On the other hand, if they show signs of misunderstanding and loss of contact, teachers reduce their pace and modify their presentation. This construction of knowledge about the classroom by the teacher is an economical strategy, because these steering groups summarize information about the impact of the action of teachers (Dahllöf, 1971; Durand, 1996; Lefeuvre and Murillo, 2017). The same is true for the students studied who categorize their teacher as authoritarian, supportive, or moody. Our results show that the students adapt their actions according to the experienced categorization (i.e., the knowledge constructed in the action about the teacher). Classroom research shows that students categorize their teachers according to a repertoire of knowledge based on significant elements of past experiences (Veyrac et al., 2018). Other work, in the field of sport, has shown that this repertoire of knowledge is mainly constructed during exploratory sequences (Sève et al., 2002, 2005). These exploratory sequences take place mainly at the beginning of the match, where the sportsman or woman seeks to know his or her opponent by testing his or her reactions and being aware of his or her interactions.

Third, the originality of our results lies in their coupling with the concerns of teachers and students. We have shown that knowledge emerges specifically according to their concerns. The teachers studied built specific knowledge coupled to their two main concerns: (re)engaging the student, exercising authority while avoiding conflict. Similarly, as regards the students studied, they constructed knowledge coupled with their concern to avoid boredom, avoid trouble with the teacher, and avoid trouble with peers. These latter results complement Allen (1986) findings showing that the strategies of students are aimed at reducing boredom and staying out of trouble during critical events. More generally, this coupling of knowledge concerns is consistent with work on difficult students leading teacher–student transactions (Flavier et al., 2002; Méard et al., 2008).

To conclude, our article gives a new point of view on knowledge during teacher–student interaction with the contribution of knowledge gained in the difficult context of vocational schools. This interactive knowledge brings to light a fine mutual knowledge that seems to be even more marked in difficult contexts (Vors et al., 2015; Kirk, 2019), and knowledge of the other and empathy seem to be very effective (e.g., Yilmaz and Sahinkaya, 2010). In recent studies, knowledge of teachers about their students is also advocated for improved student performance (Kulgemeyer and Riese, 2018; Chang et al., 2020).

Perspectives for future study could be to compare the importance given to this mutual knowledge by teachers and students, respectively, between a difficult context and a classical context; and adding outcome analysis to understand the effects of this active knowledge on learning and behavior of students. We could question the generalization of the results. On the one hand, other studies of this kind should be cross-referenced to confirm and refine our results. On the other hand, this kind of qualitative analysis then makes it possible to construct new scientific results specific to their context. The result about enacted knowledge is presented in this article, which is context dependent. The results of our study presented enacted knowledge: emerging from their context, which then raises the question of their universality. This leads us to suggest perspectives for future studies to cross different contexts of vocational high schools in France and in other countries to shed light on the question of the universality of enacted knowledge. And, with reference to the work on teacher competencies, we could consider defining teacher profiles according to the knowledge enacted in the learning situations. Finally, future research could be envisaged to study an entire class in order to validate existing knowledge categorizations and to analyze intergroup differences. We could also consider cross-referencing our qualitative data on knowledge with quantitative data *via* the measurement of the number of semantic occurrences referring to different typical knowledge in teacher–student interactions or by using self-referenced quantitative data such as the questionnaire. The articulation of these quantitative and qualitative data within the framework of mixed-methods research (Greene et al., 1989) could then shed light on enacted knowledge at the classroom level, in different contexts of vocational high schools in France and in other countries to verify the validity of the enacted knowledge presented in this study.

## Data Availability Statement

The raw data supporting the conclusions of this article will be made available by the authors, without undue reservation.

## Ethics Statement

The studies involving human participants were reviewed and approved by Aix Marseille University, ISM lab. Written informed consent to participate in this study was provided by the participants' legal guardian/next of kin.

## Author Contributions

AG and OV: conceptualization, formal analysis, investigation, methodology, and writing ± original draft. OV: project administration and supervision. All authors contributed to the article and approved the submitted version.

## Conflict of Interest

The authors declare that the research was conducted in the absence of any commercial or financial relationships that could be construed as a potential conflict of interest.
